# Modulation of NMDA Receptor Activity in Fibromyalgia

**DOI:** 10.3390/biomedicines5020015

**Published:** 2017-04-11

**Authors:** Geoffrey Littlejohn, Emma Guymer

**Affiliations:** Departments of Medicine, Monash University and Rheumatology, MonashHealth, Melbourne 3168, Australia; emma.guymer@monash.edu

**Keywords:** fibromyalgia, drugs, NMDA receptor, ketamine, memantine

## Abstract

Activation of the *N*-methyl-d-aspartate receptor (NMDAR) results in increased sensitivity of spinal cord and brain pathways that process sensory information, particularly those which relate to pain. The NMDAR shows increased activity in fibromyalgia and hence modulation of the NMDAR is a target for therapeutic intervention. A literature review of interventions impacting on the NMDAR shows a number of drugs to be active on the NMDAR mechanism in fibromyalgia patients, with variable clinical effects. Low-dose intravenous ketamine and oral memantine both show clinically useful benefit in fibromyalgia. However, consideration of side-effects, logistics and cost need to be factored into management decisions regarding use of these drugs in this clinical setting. Overall benefits with current NMDAR antagonists appear modest and there is a need for better strategy trials to clarify optimal dose schedules and to delineate potential longer–term adverse events. Further investigation of the role of the NMDAR in fibromyalgia and the effect of other molecules that modulate this receptor appear important to enhance treatment targets in fibromyalgia.

## 1. Introduction

There are multiple pain-related mechanisms that are active in patients with fibromyalgia. The relative contribution of each mechanism appears to vary between patients. This results in different responses to different drugs in different patients suggesting that the symptoms that characterize fibromyalgia need to be targeted through a number of different approaches [1].

One important mechanism in fibromyalgia is enhanced reactivity in a number of sensory systems, particularly the pain-related nervous system [2,3]. Of importance is the interaction between the peripheral mechanoreceptors and the deep spinal cord neurons that relay sensory information to regions of the brain that relate to the perception of pain [4]. Through these processes, low-level non-noxious stimuli that activate mechanoreceptors in structures such as muscles, tendons, ligaments and entheses, will be perceived as painful. The mechanism that promotes the interaction between the mechanoreceptor input and the deeper pain-related spinal cord neurons is key to the understanding of fibromyalgia.

Increased sensitivity of spinal cord neurons occurs in fibromyalgia [2,4]. This involves those that are involved in reception of nociceptive input, involving C- and A-Δ fibres, as well as those more deeply placed polymodal neurons that are able to receive mechanoreceptor input. These processes in turn are dependent on the function of the *N*-methyl-d-aspartate receptor (NMDAR) [4,5,6].

This clinical review examines publications that relate to NMDAR modulation in patients with fibromyalgia. 

## 2. Methods

We performed a search of PubMed using the following keywords in various combinations: “fibromyalgia, NMDA, ketamine, memantine, mechanisms, glutamate”. We felt that PubMed would provide a comprehensive collection of studies on fibromyalgia. The bibliography of relevant identified papers was surveyed and information from abstracts and on-line sources was included as deemed relevant. We supplemented this information with that contained in the authors’ own databases.

## 3. Results

### 3.1. NMDAR Function in Fibromyalgia

Various imaging techniques have shown elevated glutamate levels in the brains of patients with fibromyalgia [7]. For instance, elevated glutamate (measured with glutamine) is seen in the posterior insula in fibromyalgia patients, with positive correlations with lower pain thresholds, a characteristic feature of fibromyalgia [8]. Further studies showed that depletion of glutamate levels by pregabalin predicted the subsequent analgesic response to this drug [9]. Additionally, brain spectroscopy studies have shown a strong correlation between high levels of glutamate in the posterior cingulate and pain in fibromyalgia [10]. As well as patients with fibromyalgia having increased glutamate in pain-related brain regions [11] , there is also increased glutamate and glycine in cerebrospinal fluid in fibromyalgia patients [12]. Hence NMDA receptors in the spinal cord and brain of patients are exposed to increased levels of glutamate. Mechanisms that relate to descending control of spinal neuron function are abnormal in fibromyalgia and these changes may also interact with function of the NMDAR at the spinal cord level. 

**Figure 1:**
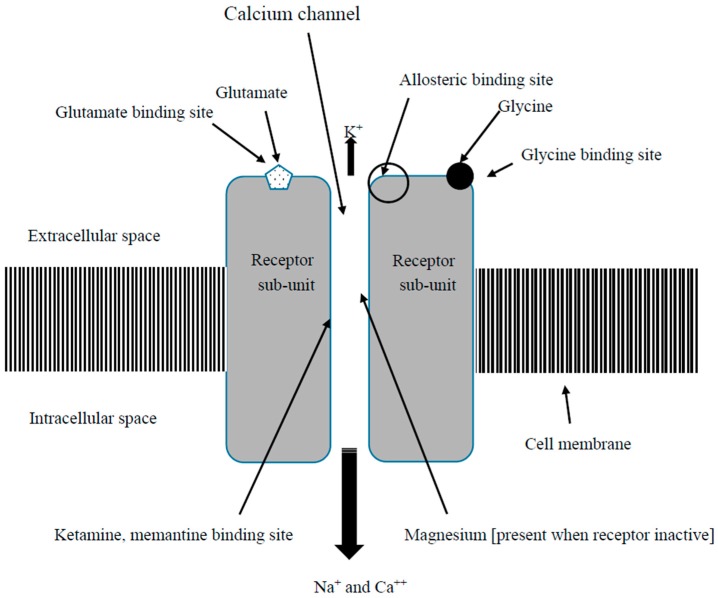
Simplified diagram of activated *N*-methyl-d-aspartate receptor (NMDAR) showing sites where key molecules interact.

The amino acid glutamate, acting on two different groups of receptors (labeled ionotropic and metabotropic), is primarily involved in excitatory synaptic transmission in the brain and spinal cord. Ionotropic glutamate receptors are ligand-gated ion channels. They are divided into different receptor types based on their pharmacological properties. The key receptor sub-groups are GluA (AMPA, 2-amino-3-3-hydroxy-5-methyl-isoxazol-4-yl propanoic acid), GluD (δ), GluK (kainate), and the GluN (NMDA, *N*-Methyl-d-aspartic acid) [13]. The NMDAR (Figure 1), named because the agonist molecule NMDA binds specifically to it and not to other glutamate receptors, plays a key role in neural plasticity as well as excitotoxicity [13,14]. In its normal resting state the receptor is inactive and does not participate in synaptic modulation. This is because the ion channel is “plugged” by the binding of extracellular magnesium and zinc that impede the flow of cations through the channel [15]. The NMDAR is activated through the binding of the ligands glutamate and glycine (or d-serine) to different sites of the NMDAR. Cellular depolarization repels the magnesium and zinc ions from the channel allowing voltage dependent entry of sodium and small amounts of calcium into the cell and egress of potassium out of the cell. This process is primarily gated by ligand binding, but is also voltage dependent [13]. Thus an NMDAR that has glycine and glutamate bound to it and has an open channel to allow electrical signals to pass is deemed “activated”. Adequate glycine is thought to be always present in the synaptic gap but glutamate levels relate to release from presynaptic terminals. Hence, activation of these receptors links to glutamate availability at the receptor level. The function, structure and distribution of NMDARs through the brain, spinal cord and peripheral tissues show considerable heterogeneity, with variation of subunits, including NR1 and NR2 [14,16,17]. 

NMDAR antagonists that inhibit activation by agonists to the NMDAR fall into four categories. These comprise competitive antagonists, which bind to and block the binding site of either the neurotransmitter glutamate or that of glycine, noncompetitive antagonists, which inhibit NMDARs by binding to allosteric sites, and other antagonists, which block the ion channel by binding to a site within the channel [18].

### 3.2. NMDAR Inhibitors

#### 3.2.1. Ketamine

Ketamine 2-(2-chlorophenyl)-2-(methylamino)-cyclohexanone) is a non-competitive NMDAR antagonist. The *S* (+) stereo-isomer is the most potent NMDAR-antagonist in clinical use, and is 3–4 times that of the *R* (-) isomer. Ketamine binds to an intrachannel site and decreases channel opening time [19]. Ketamine also acts on opioid, non-NMDA glutamate and muscarinic cholinergic receptors, and facilitates GABA signaling, and has local anesthetic properties. Ketamine is bound with greater affinity to agonist sites on high-affinity dopamine D2 receptors than to NMDARs [20]. Thus the clinical effects of ketamine may relate to a number of actions and not just those on the NMDAR [21]. The common side-effects of nausea, headache, dizziness and confusion relate to the various actions of ketamine. Ketamine also has significant psychomimetic effects that influence its clinical utility [15,22].

While ketamine has been widely used in the management of a number of chronic pain disorders [23] there are few studies showing long term benefit [22]. Ketamine has been shown to be effective in severe major depressive disorder which may be present in some patients with fibromyalgia [24]. 

The clinical effects of ketamine have been evaluated in a small sample of patients with fibromyalgia. A double-blind study of 11 female patients with fibromyalgia given low-dose ketamine infusions (0.3 mg/kg) or sodium chloride (placebo) at different times over a period of 0 to 10 min in a random cross-over design evaluated a number of relevant outcome measures [25]. Pain intensity change of <50% was labeled as placebo response. One patient was a placebo responder, 8 were deemed ketamine responders and 2 non-responders. There was a significant reduction in pain intensity (>50%) with the ketamine infusion compared to the saline infusion during (*p* < 0.05) and 20–80 min after the test period (*p* < 0.01). There was a decrease in tenderness (*p* < 0.02) and increased endurance (*p* < 0.02). The change in pain threshold and pain tolerance at tender points (<0.02 and <0.0001 respectively) and control points (<0.03 and <0.02 respectively) were each significant. Six of the 8 responders had reduction in pain for 2 to 7 days. In addition to the 11 ketamine-infused patients, 9 other patients were treated with morphine and compared to saline (no significant change in the above outcomes) and 11 other patients were treated with lidocaine and compared to saline (pain decrease during and after for short time after the infusion, *p* < 0.05).

These studies were extended, using saline, lidocaine, morphine and ketamine, in a total of 18 patients [26]. Thirteen patients responded to one or several of the drugs; 2 were placebo responders to all 4 infusions, and 3 patients did not respond to any infusion. Seven of the responders had pain reduction for 1 to 5 days. The 8 responders to ketamine significantly improved Fibromyalgia Impact Questionnaire (FIQ) scores. Blood drug levels were the same in responders and non-responders. 

A third study using similar methodology identified 17 of 29 fibromyalgia patients as responders to ketamine [27]. Thus, of 58 patients with fibromyalgia in the above 3 studies, 33 (57%) responded to low dose ketamine (0.3 mg/kg) infusion, as defined by a reduction of pain by 50% or more [28].

A subsequent study assessed the effect of either placebo or ketamine on pain induced by intramuscular infusion of hypertonic saline in patients with fibromyalgia who had previously been defined as ketamine responders. These studies showed significant parallel reduction in pain intensity, temporal summation, allodynia and area of referred pain in those given the NMDAR-antagonist ketamine compared to those given placebo [27]. 

Taken as a whole, these described studies imply that NMDAR activation significantly contributes to the pathophysiology of the pain of fibromyalgia. However, the short time period of observation in these studies in a chronic pain condition such as fibromyalgia limits the clinical usefulness of this data.

A double-blind placebo controlled trial in 24 fibromyalgia patients examined durability of response to ketamine by comparing a single infusion of low dose (0.5 mg/kg) *S*-ketamine to an infusion of 5 mg midazolam, each over 30 min [29]. This showed that the initial significant pain reduction by ketamine, compared to midazolam, waned quickly in parallel with the pharmacokinetics of ketamine. There was no difference between *S*-ketamine treated patients and midazolam treated patients at 2.5 h after the infusion or during the 8-week follow-up time period. Psychomimetic side effects were mild to moderate and similar between groups. The *S*-ketamine provided a dose that was 2.5 times higher than the racemic ketamine used in the studies of Sorenson [26] and Graven-Nielsen [27] but the duration of effect was similar suggesting that the duration of infusion may be a critical factor in achieving durable and clinically meaningful pain reduction.

Complex regional pain syndrome (CRPS) shares many pathophysiological features with fibromyalgia [30], including response to ketamine. In a study of 60 patients with CRPS 4 days of continuous low-dose (up to mean of 22 mg/h/70 kg) ketamine infusion resulted in clinically relevant pain reduction in the ketamine treated group, compared to the placebo treated group, lasting for 11 weeks [31]. The effect was not influenced by duration of CRPS but the pain relief was not accompanied by improved functional status.

Anecdotal contemporary use of intravenous ketamine for the pain of fibromyalgia generally involves doses that are higher, of longer duration and of greater frequency than in the initial clinical studies. For instance, the dose may start at 200 mg over 4 h on day 1, 600 mg over 4 h on day 2, and 800 mg over 4 h on day 3, 4 and 5, with a booster of 800 mg over 4 h on each of two days some two weeks later [32]. The dose escalation is tempered by possible side effects. Diazepam and ondansetron are often given at the time of infusion to minimize common side effects of agitation and nausea. Psychomimetic effects may moderate the dosing schedule.

There are no randomized controlled studies of this higher dose and longer duration use of ketamine in fibromyalgia.

The use of oral ketamine for treatment of fibromyalgia has not been widely studied, however one study reported clinically meaningful responses (>50% pain reduction) in a small number of patients [33]. If effective the oral ketamine was usually continued for 3 months as the adverse effects of long-term use of ketamine were considered to be unclear [33]. The mean effective dose in the whole study group (which included the fibromyalgia patients) was 2mg/kg after titration. Adverse events were generally mild and resulted in drug cessation in only a small number of patients. It has been noted that the oral route is associated with fewer side effects than the parenteral route [34,35]. It is noted that when administered orally, a metabolite of ketamine can contribute to its actions [19].

Special issues arise regarding recreational use of this drug and these need to be considered when planning future studies in fibromyalgia and other pain disorders [36].

#### 3.2.2. Dextromethorphan

Dextromethorphan is often present in over-the-counter cough medications and has mild NMDAR non-competitive channel blocking actions [37].

The response to an intravenous low-dose (0.1 mg/kg) ketamine infusion in 34 patients with fibromyalgia predicted the subsequent response to the oral NMDAR-antagonist dextromethorphan (mean dose in responders 160 mg/day), although 56% of patients responded to neither drug [38]. The value for a positive response to the intravenous ketamine test was established at 67% pain relief, and a positive response to the dextromethorphan treatment was defined as a 50% reduction at 4–6 weeks after treatment was commenced. The degree of correlation between pain relief with ketamine and dextromethorphan was highly significant (*p* < 0.001). There was a statistically significant association between the occurrences of side effects in each group. Ketamine side effects included dizziness, confusion, euphoria or a combination of these. Dextromethorphan related side effects included dizziness and sedation.

In a study of fibromyalgia patients compared to healthy controls, there was a similar response to the NMDAR antagonist dextromethorphan when assessed using the effects of temporal summation of dorsal horn neuronal responses, which reflects nociception-dependent central sensitization [6]. This suggests that NMDAR-related pain mechanisms may be dominant or responsive to modulation in only a sub-set of patients with fibromyalgia, which is consistent with the clinical observations with various NMDAR-antagonists. 

#### 3.2.3. Memantine

Memantine is a non-competitive blocker of the NMDAR channel that leads to reduction of glutamate and prevents entry of excess calcium [39]. It dissociates from the channel and thus decreases pathological activity of the NMDAR without changing normal synaptic function [39]. Memantine has a low side-effect profile and can be used over a prolonged period of time [40]. It has been effective in complex regional pain syndrome [41], a condition that shares many pathophysiological features with fibromyalgia [30].

A randomised, double-blind study in 63 patients with fibromyalgia compared memantine (titrated up to 20 mg/day over one month) with placebo over a 6-month period [40]. Compared to placebo there was a significant reduction in pain and increase in pain threshold, and improvement in global function, mood and quality of life. Compared to placebo, and using decrease in pain intensity of 50% as the end-point, the absolute risk reduction was 16% and the number needed to treat to achieve that end-point was 6.2. Dizziness and headache occurred in a minority.

In an associated study, it was shown that patients treated with memantine showed significant changes in brain glutamate-related metabolites compared to placebo-treated patients [42]. There was a correlation between choline levels and the FIQ score in the posterior insula.

Table 1 summarizes the doses used and side-effects noted from the reported use of ketamine, dextromethorphan and memantine in fibromyalgia.

#### 3.2.4. Amantadine

Amantadine is a weak non-competitive NMDAR channel blocker [43] that has shown variable results in neuropathic pain [44] but has not been studied in fibromyalgia. Amantadine, usually taken orally at 100 to 200 mg per day, produces hypotension, dizziness, agitation, confusion and hallucinations. Anecdotal use suggests limited benefit in fibromyalgia. 

#### 3.2.5. Methadone

Methadone is a μ-opioid receptor agonist that also has NMDAR antagonist activity [45]. It is used in opioid withdrawal programs. It has not been studied in fibromyalgia but it has been shown to have effect in patients with neuropathic pain. In a small uncontrolled observational study of 18 patients with neuropathic pain a mean stable dose of 15 mg per day associated with a significant reduction in mean pain levels (VAS +/− SD of 7.7+/− 1.5 to 1.4+/− 1.7, *p* < 0.0001), and 70 percent had complete resolution of mechanical allodynia [46]. It is noted that the dextrorotatory form (d-methadone) acts as a NMDAR antagonist without opioid activity. 

There is little role for the use of pure opioids in fibromyalgia as intrinsic brain opioid activity is already optimized and this, together with the significant medical issues associated with long-term opioid use, limits consideration of the usual form of this drug for its NMDAR antagonist properties in fibromyalgia [47]. This drug should only be considered in special circumstances in fibromyalgia, for instance as part of an opioid withdrawal program. 

#### 3.2.6. Guaifesin

Guaifesin, a drug with expectorant properties, may also have NMDAR antagonist actions [48]. However, a trial comparing the use of guaifesin to placebo in two groups of 20 patients with fibromyalgia over a 12–month observational period showed no difference in outcomes [49].

### 3.3. Drugs with Indirect Effect on NMDAR Function

A number of drugs used in the management of fibromyalgia, such as different antidepressants, likely have an indirect effect on NMDAR function through reduction of the NMDAR ligands glutamate and aspartate [50,51]. Glutamate reduction in relevant pain-related brain regions in fibromyalgia patients is seen with pregabalin [9,52], with beneficial clinical effects likely to involve changes in NMDAR function in brain and spinal cord due to less synaptic glutamate being available to facilitate receptor activation. Pregabalin also decreases synaptic substance P and noradrenaline, and has other central effects relevant to fibromyalgia management, including improvement in sleep quality and anxiety [53]. The clinical effects of these drugs in fibromyalgia are not the main subjects of this review.

### 3.4. Non-Pharmacological Approaches

Dietary modification of foods high in glutamate has been trialed in fibromyalgia. One study showed that 4 weeks of exclusion of monosodium glutamate (MSG), aspartame, and other excitotoxins, resulted in over 30% improvement in fibromyalgia symptoms in 84% of those who completed the diet [54]. When active MSG was added back in the diet, there was a return of fibromyalgia symptoms in a significant proportion of patients compared to an inactive placebo. This suggests that dietary glutamate may modulate fibromyalgia symptoms in some patients.

### 3.5. NMDA Antagonists in Fibromyalgia Management Guidelines

Drugs that are active against NMDARs are either not mentioned or not recommended in a number of evidence-based guidelines and reviews for management of fibromyalgia [55,56,57]. Some guidelines indicate the unproven potential of targeting the NMDAR in fibromyalgia [58].

## 4. Conclusions

The NMDAR plays a prominent role in the pathophysiology of fibromyalgia. A number of drugs that target and down-regulate this structure cause a reduction of fibromyalgia-related symptoms. While this review provides evidence for the participation of NMDARs in the mechanism of fibromyalgia, it also highlights limitations in methodology of the identified studies. These are characterized by several short duration studies in a condition that is usually long-standing. Additionally, there are few studies examining different doses of NMDAR antagonists and none assessing combinations with drugs of different classes. Further study of currently available NMDAR antagonist drugs and assessment of new drugs in this class is warranted.

## Figures and Tables

**Table 1:** Selected NMDAR antagonists that have been used in the treatment of fibromyalgia.

Drug	Analgesic Dose	Side Effects	Comment
Ketamine	Oral: 2 mg/kg IV: 0.2–0.75 mg/kg Continuous infusion: 2–7 mcg/kg/min	Psychomimetic—hallucinations, confusion, sedation, irrational behaviour	No studies of higher dose, longer duration regimens limit use.
Dextromethorphan	Oral: 45–400 mg/day	Drowsy, dizzy, anxiety, confusion	Few clinically useful studies, anecdotal use suggests limited effect.
Memantine	Oral: 10–30 mg/day	Hypertension, dizzy, drowsy, nausea, anxiety, hallucinations	Further studies may show this drug to be clinically useful.
